# Aging Increases Short, Inverted Repeat‐Mediated Genomic Instability In Vivo

**DOI:** 10.1111/acel.70250

**Published:** 2025-09-30

**Authors:** Pooja Mandke, Pallavi Kompella, Guliang Wang, Karen M. Vasquez

**Affiliations:** ^1^ Dell Pediatric Research Institute, Division of Pharmacology and Toxicology College of Pharmacy, the University of Texas at Austin Austin Texas USA

**Keywords:** aging, cruciform DNA, DNA damage, genomic instability, inverted repeats, mutagenesis

## Abstract

Genomic instability is a hallmark of aging and cancer. A key contributor to genomic instability includes alternative DNA structures, such as cruciform‐forming inverted repeats (IRs). Short IRs (< 100 bps) are abundant in the human genome, mutagenic, and enriched at mutation hotspots in human cancer genomes. Using an innovative mutation‐reporter mouse model, we showed that short IRs are mutagenic in vivo. Further, we found that aging exacerbates IR‐induced genomic instability, as evidenced by increased mutation frequencies and altered spectra in the spleen and brain of mice harboring either a short IR or control B‐DNA sequence at 2 and 24 months of age. These findings establish a link between aging and enhanced mutagenesis at short IRs, providing a unique in vivo platform to investigate age‐related mechanisms of DNA structure‐mediated genomic instability.

## Graphical abstract

Aging amplifies the mutagenic potential of short, inverted repeats in mice. The increase in point mutations in the spleen and large deletions in the brain with age highlights distinct pathways across proliferative and post‐mitotic tissues.

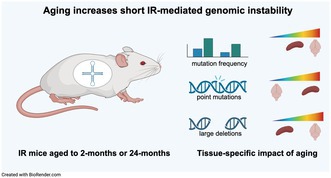

## 1 Introduction, Results, and Discussion

Over the last century, rising life expectancy (Butler 2012) has been accompanied by an increase in cancer incidence (Siegel et al. 2024). According to the National Cancer Institute, cancer rates increase sharply with age, exceeding 1000 cases per 100,000 in those over 60 (Siegel et al. 2021). Therefore, understanding the mechanisms that link aging and disease development is critical.

**FIGURE 1:**
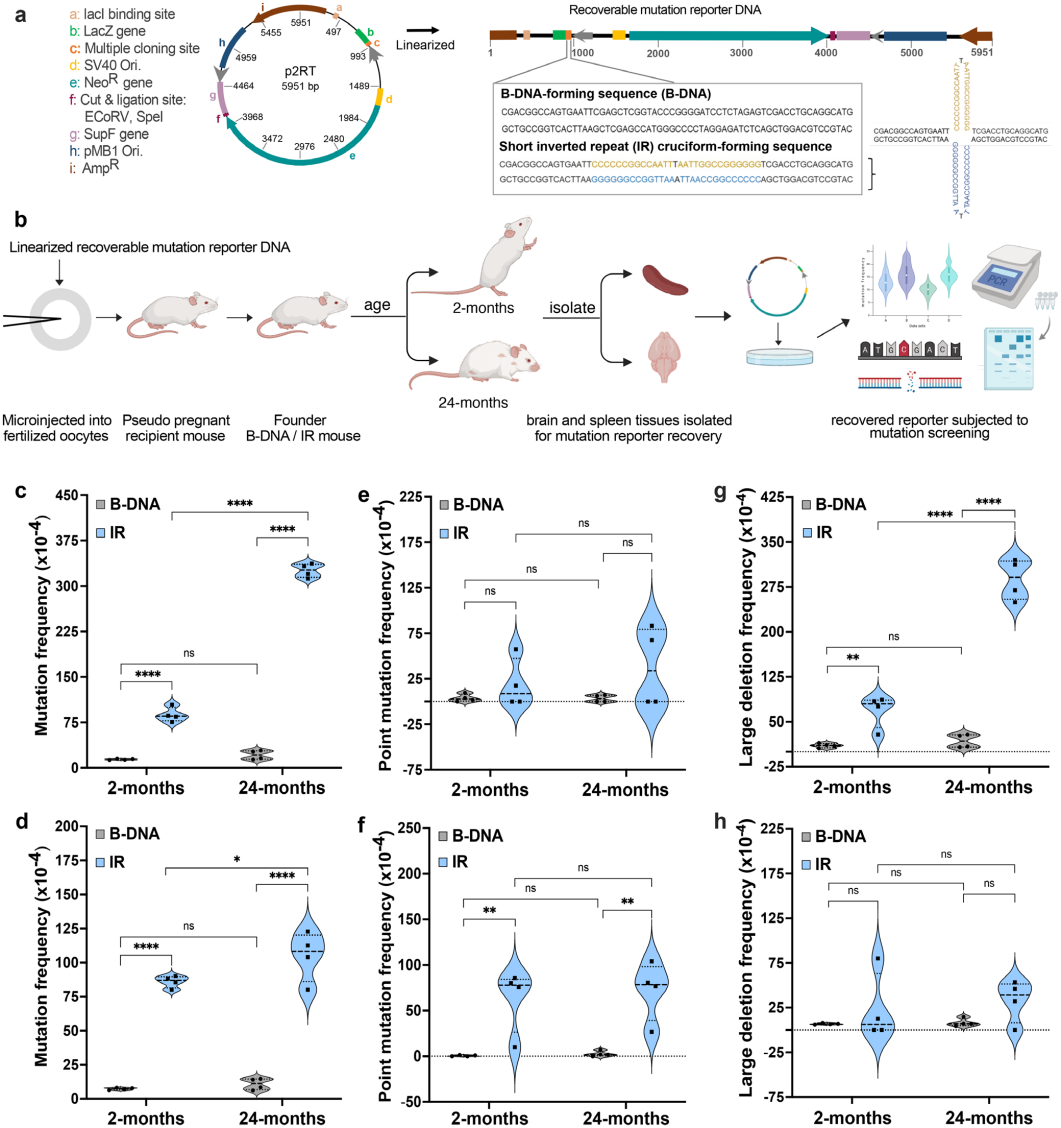
Aging increases IR‐induced genomic instability in a tissue‐specific manner. (a) Schematic of the p2RT mutation reporter, containing either a 29‐bp canonical B‐DNA structure‐forming sequence or a 29‐bp cruciform structure‐forming inverted repeat (IR) sequence. (b) Groups of male and female FVB/N mice (*n* = 4/group) containing the linearized mutation reporter with a B‐DNA‐forming or a cruciform‐forming IR sequence in their chromosomes were aged for 2 and 24 months. Mutation reporters recovered from brain and spleen tissues were screened for mutation frequency using a lacZ blue‐white mutagenesis assay and categorized as point mutation and large deletion frequencies. (c) Mutation frequency (x10^‐4^) in brain tissue, interaction (F(1,12) = 642, ****, *p* < 0.0001), age factor (F(1,12) = 726, ****, *p* < 0.0001), B‐DNA versus IR factor (F(1,12) = 1724, ****, *p* < 0.0001). (d) Mutation frequency (x10^‐4^) in spleen tissue, interaction (F(1,12) = 2.55, ns, *p* = 0.13), age factor (F(1,12) = 5.41, *, *p* = 0.038), B‐DNA versus IR factor (F(1,12) = 323.1, ****, *p* < 0.0001). (e) Frequency of point mutations (x10^‐4^) in brain tissue, interaction (F(1,12) = 0.55, ns, *p* = 0.47), age factor (F(1,12) = 0.51, ns, *p* = 0.49), B‐DNA versus IR factor (F(1,12) = 3.6, ns, *p* = 0.08). (f) Frequency of point mutations (x10^‐4^) in spleen tissue, interaction (F(1,12) = 0.08, ns, *p* = 0.77), age factor (F(1,12) = 0.21, ns, *p* = 0.65), B‐DNA versus IR factor (F(1,12) = 29.79, ***, *p* = 0.0001). (g) Frequency of large deletions (x10^‐4^) in brain tissue, interaction (F(1,12) = 87.8, ****, *p* < 0.0001), age factor (F(1,12) = 101, ****, *p* < 0.0001), B‐DNA versus IR factor (F(1,12) = 212, ****, *p* < 0.0001). (h) Frequency of large deletions (x10^‐4^) in spleen tissue, interaction (F(1,12) = 0.13, ns, *p* = 0.72), age factor (F(1,12) = 0.24, ns, *p* = 0.63), B‐DNA versus IR factor (F(1,12) = 3.28, ns, *p* = 0.095). Data for the biological replicates from B‐DNA mice (2‐months of age, *N* = 4; 24‐months of age, *N* = 4) and IR mice (2‐months of age, *N* = 4; 24‐months of age, *N* = 4) are represented as violin plots with dotted lines indicating the median and quartile. Data was analyzed using two‐way ANOVA for significance in interaction (age × B‐DNA/IR) followed by Sidak multiple comparison test for age factor (2‐months of age vs. 24‐months of age) and B‐DNA versus IR factor. The icons in panel b were created with BioRender.com and released under a Creative Commons Attribution‐NonCommercial‐NoDerivs 4.0 International license.

Genomic instability is a hallmark of both aging and cancer, contributing to the accumulation of mutations that drive disease progression (Aunan et al. 2017; Maslov and Vijg 2009; Patel et al. 2020). While various extrinsic factors such as environmental stressors contribute to genomic instability, repetitive DNA sequences capable of adopting alternative DNA structures represent a significant intrinsic source (G. Wang and Vasquez 2006, 2023; Zhao et al. 2010). These sequences are enriched at mutation hotspots in human cancer genomes, implicating them in cancer etiology (Bacolla et al. 2016). One type of repetitive sequence, inverted repeats (IRs), can undergo intra‐strand base pairing, forming hairpins (Nag and Petes 1991) when a single DNA strand folds back, or cruciform structures when both strands participate, forming a four‐way junction (Figure 1a).

Cruciform structures play functional roles in DNA replication, transcription, recombination, gene expression, and genome organization (Brazda et al. 2011; Dai et al. 1997; Shlyakhtenko et al. 2000), and also contribute to genomic instability in bacteria, yeast, and mammalian cells (Cromie et al. 2000; Henderson and Petes 1993; Kato et al. 2008; Lu et al. 2015; Nasar et al. 2000; Tanaka et al. 2002). In addition, cruciform‐forming IRs have been implicated in cancer‐associated chromosomal translocations (Kurahashi et al. 2004) and in Werner syndrome, a premature aging disorder (Brazda et al. 2011).

Much research has focused on long IRs (> 500 bp), which promote deletions in bacteria and yeast (Weston‐Hafer and Berg 1991; Nasar et al. 2000). Although rare in the human genome (Y. Wang and Leung 2006), long palindromic AT‐rich repeats (PATRRs) enriched near breakpoints on chromosomes 11 and 22 can form cruciform structures. If resolved improperly, they can cause DNA double‐strand breaks (DSBs) (Correll‐Tash et al. 2021; Edelmann et al. 2001; Kurahashi et al. 2004), stimulating recurrent chromosomal translocations and resulting in a congenital disorder called Emanuel syndrome (Correll‐Tash et al. 2021; Zackai and Emanuel 1980).

In contrast, short IRs (≥ 8 bp), which are the focus of this study, are abundant in the human genome (~1 per 5600 bp) (Lu et al. 2015; Schroth and Ho 1995), with ~80% being less than 100 bp in length (Y. Wang and Leung 2006). We have previously shown that short (29 bp) perfect IRs can induce DSBs and deletions in yeast and mammalian cells via replication‐dependent fork stalling or replication‐independent cleavage by ERCC1‐XPF (Lu et al. 2015). Importantly, short IRs are enriched at translocation breakpoints in human cancer genomes, implicating them in cancer etiology (Bacolla et al. 2016).

Interestingly, hairpins formed at triplet repeats have shown age‐ and tissue‐specific instability (Hubert Jr. et al. 2011; Kovtun et al. 2007); prompting us to investigate whether aging also influences cruciform‐mediated genomic instability in a tissue‐specific manner. We assessed the targeted effects of aging using transgenic mice (D'Amico et al. 2024; Kompella et al. 2024; G. Wang et al. 2008) with chromosomally integrated mutation reporters containing a control B‐DNA‐forming sequence (B‐DNA mice) or a short IR cruciform‐forming sequence (IR mice) (Figure 1a,b). Groups (*N* = 4) of male and female B‐DNA and IR mice were aged to 2 or 24 months (Figure 1b). Mutation reporter DNA was recovered from the brain (low proliferation) and spleen (high proliferation) tissues to assess age‐associated mutation patterns by blue‐white mutagenesis analyses (G. Wang et al. 2009) (Figure S1). Additionally, large deletion analysis was performed by PCR. Across both tissues, IR mice showed significantly higher mutation frequencies than B‐DNA mice at both ages (*p* < 0.0001). In the brain tissue, mutation frequencies were significantly higher in the IR mice compared to B‐DNA mice at both 2 and 24 months of age (*p* < 0.0001) (Figure 1c). At 2 months, IR mice showed a ~6‐fold increase in mean mutation frequency over B‐DNA mice (87.6 × 10^−4^ vs. 13 × 10^−4^, *p* < 0.0001), which increased to ~14‐fold at 24 months (325.6 × 10^−4^ vs. 21.2 × 10^−4^, *p* < 0.0001). Aging led to a ~4‐fold increase in mutation frequencies in IR mice (325.6 × 10^−4^ vs. 87.6 × 10^−4^, *p* < 0.0001), while B‐DNA mice showed no significant age‐related changes (21.2 × 10^−4^ vs. 14 × 10^−4^, *p* = 0.48). Similarly, in the highly proliferative spleen tissue (Bronte and Pittet 2013), IR mice showed significantly higher mutation frequencies than B‐DNA mice at both ages (*p* < 0.0001) (Figure 1d). IR mice had a ~12‐fold increase (86 × 10^−4^ vs. 7.3 × 10^−4^, *p* < 0.0001) and a ~9.6‐fold increase (104.9 × 10^−4^ vs. 10.8 × 10^−4^, *p* < 0.0001) over B‐DNA mice at 2 months and 24 months, respectively. A modest but significant age‐related increase of 1.2‐fold was seen in IR mice (104.9 × 10^−4^ vs. 86 × 10^−4^ *p* = 0.03), whereas a similar effect of age was not observed in B‐DNA mice (10.8 × 10^−4^ vs. 7.3 × 10^−4^, *p* = 0.85).

The dramatic increase in IR and age‐driven mutation frequencies in the brain may stem from a rise in oxidative stress with aging, which can accelerate the accumulation of mutations in non‐replicative cells. In contrast, age‐associated decline in base excision repair efficiency could further lead to elevated mutagenesis (Imam et al. 2006). The observation in our short IR model, which forms perfect hairpin structures on both strands (i.e., cruciform), is consistent with prior reports for triplet repeats forming hairpins (Hubert Jr. et al. 2011; Kovtun et al. 2007).

Interestingly, we observed distinct mutation spectra across both tissues. In brain tissue, point mutation frequencies did not differ significantly between IR and B‐DNA mice at 2 months (18.7 × 10^−4^ vs. 3.7 × 10^−4^, *p* = 0.68) or 24 months (37.6 × 10^−4^ vs. 3.32 × 10^−4^, *p* = 0.1) (Figure 1e, Table S1). Further, there was no significant effect within the two age groups of IR and B‐DNA mice, suggesting that point mutations may arise through a shared mechanism that is independent of aging. In contrast, at 2 months, spleen tissue showed a remarkable ~106‐fold increase in IR‐associated point mutations over B‐DNA mice (62.8 × 10^−4^ vs. 0.6 × 10^−4^, *p* = 0.006), which remained elevated at 24 months with a ~26‐fold difference (71.9 × 10^−4^ vs. 2.6 × 10^−4^, *p* = 0.003) (Figure 1f, Table S2).

In addition to point mutations, a few small deletions (< 30 bp) were detected (Table S1) in the brain tissue but were rare and showed no clear age‐ or sequence‐specific trends. In contrast, large deletions (> 3000 bp) were significantly elevated in the brain tissue of IR mice. At 2 months, we observed a ~7‐fold increase in large deletion frequency in IR mice compared to B‐DNA mice (68.9 × 10^−4^ vs. 10.3 × 10^−4^, *p* = 0.0063), which increased to ~17‐fold by 24 months (288.9 × 10^−4^ vs. 17.9 × 10^−4^, *p* < 0.0001) (Figure 1g, Table S1). The impact of age was also evident, with a ~4‐fold increase from 2 to 24 months in IR mice (287.9 × 10^−4^ vs. 68.8 × 10^−4^, *p* < 0.0001), with no significant age‐related change in B‐DNA mice (17.2 × 10^−4^ vs. 10.2 × 10^−4^, *p* = 0.87). These results suggest that large deletions are the dominant mutation type induced by short IRs in the brain tissue, and their frequency increases in an age‐dependent manner. Interestingly, the age‐dependent increase in IR‐induced large deletions was not observed in the spleen tissue. Large deletion frequencies did not differ significantly between IR and B‐DNA mice at either 2 months (23.1 × 10^−4^ vs. 6.7 × 10^−4^ *p* = 0.54) or 24 months (32.9 × 10^−4^ vs. 8.2 × 10^−4^ *p* = 0.27) (Figure 1h, Table S2). Additionally, no significant age‐related changes were detected within the two age groups of IR and B‐DNA mice. These results suggest that brain‐specific metabolic features may contribute to IR‐induced large deletions with aging.

The predominance of large deletions in the brain tissue may be attributed to age‐related changes in DNA repair efficiency. Although several proteins are known to interact with cruciform DNA structures (Bowater et al. 2022; Brazda et al. 2011; Lu et al. 2015), their roles in mutagenic processing are not fully understood. Thus, tissue‐specific differences in DNA repair capacity could contribute to the observed age‐dependent short IR‐induced genomic instability. These differences may reflect distinct mechanisms by which alternative DNA structures are processed. In the low‐proliferating brain tissue (Reu et al. 2017), cruciform structures may be recognized and cleaved by structure‐specific endonuclease ERCC1‐XPF, generating DSBs and large deletions in a replication‐independent manner (Lu et al. 2015; McKinney, Wang, and Vasquez 2020). In contrast, in the highly proliferative spleen tissue (Richardson et al. 2014), cruciform structures may stall replication forks, leading to the recruitment of low‐fidelity translesion synthesis DNA polymerases, which can introduce mutations in a replication‐dependent manner (Lu et al. 2015; McKinney, Wang, and Vasquez 2020). However, unresolved fork stalling can lead to fork collapse, resulting in DSBs and large deletions, as observed in a subset of spleen mutants across both age groups.

**FIGURE 2:**
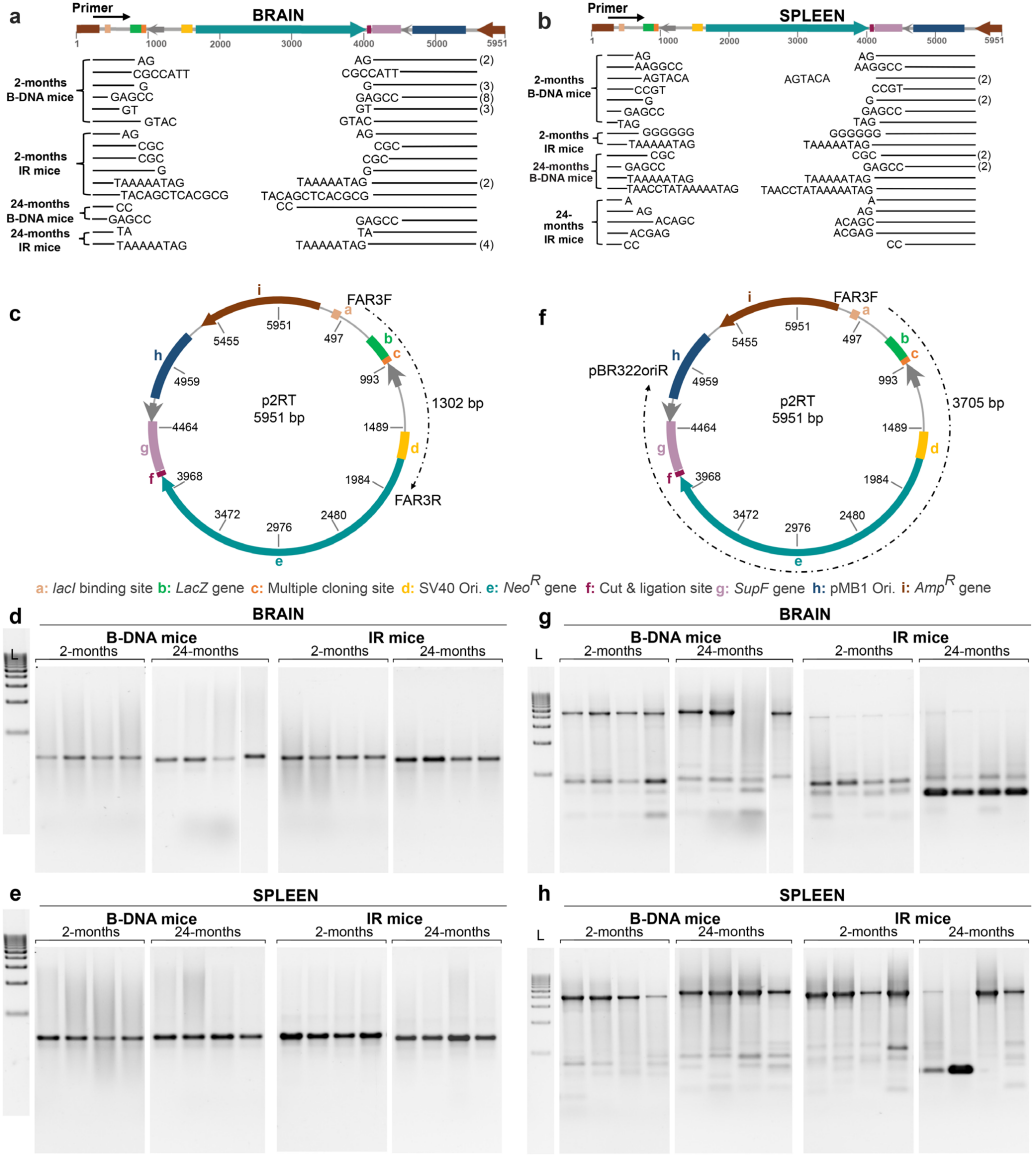
Aging increases IR‐induced large deletions in a tissue‐specific manner. (a) Deletion mutations were mapped for position and size of deletion by comparing reporters recovered from brain tissue (*n* = 32) and (b) spleen tissue (*n* = 22), from B‐DNA mice (2‐months of age, *N* = 4; 24‐months of age, *N* = 4) and IR mice (2‐months of age, *N* = 4; 24‐months of age, *N* = 4) against the linearized p2RT mutation reporter sequence. The blank regions between the lines represent deletions, while the bases at the ends of the lines denote microhomologies at the deletion junctions. A single copy of microhomology is present at the deletion junction but is shown on both ends, as it cannot be definitively assigned to either side. The number of identical deletion mutants is indicated in parentheses to the right. (c) Schematic of the mutation reporter with regions amplified by the FAR3F/FAR3R primers producing a 1302 bp PCR product. (d, e) PCR products amplified using the FAR3F/FAR3F primers on genomic DNA isolated from brain tissue and spleen tissue, respectively, of 2‐month‐old and 24‐month‐old B‐DNA and IR mice. (f) Schematic of the mutation reporter with regions amplified by the FAR3F/pBR322OriR primers producing a 3705 bp PCR product. (g, h) PCR products amplified using the FAR3F/pBR322OriR primers on genomic DNA isolated from brain tissue and spleen tissue, respectively, of 2‐month‐old and 24‐month‐old B‐DNA and IR mice respectively. Amp^R^, ampicillin resistance; Neo^R^, neomycin resistance; Ori, origin of replication.

Sanger sequencing of the large deletions (> 3000 bp) in both B‐DNA and IR mice revealed microhomologies at the breakpoint junctions in the brain (Figure 2a) and spleen tissues (Figure 2b). These results suggest the repair of DSBs via microhomology‐mediated end‐joining (MMEJ), a pathway we and others have linked to alternate DNA structure processing (Kha et al. 2010; Lu et al. 2015; McKinney, Wang, Mukherjee, et al. 2020; Zhao et al. 2018). The consistent presence of microhomologies, regardless of tissue type, provides further evidence for the role of MMEJ in processing IR‐associated breaks. Notably, the increased reliance on MMEJ with age in brain tissue may be linked to a decline in classical non‐homologous end‐joining (NHEJ) efficiency, as observed in other post‐replicative tissues, such as the heart (Vaidya et al. 2014).

To detect mutations in the mouse genome, the lacI‐recovery reporter system provides a sensitive and facile assay capable of detecting rare mutational events at frequencies as low as ~10^−5^. This level of sensitivity allows for the robust quantitative assessment of a broad spectrum of mutation types (base substitutions, small insertions/deletions, and complex mutations) across multiple tissues with high reproducibility. However, successful detection in this system relies on two key factors: (1) the presence of at least one of the two lacI binding sites, one located within the *lacZ* promoter and the other located 595 bp downstream; and (2) the integrity of the ampicillin resistance gene and the origin of replication. Given the high number of mutants observed, particularly in the brain tissue, we considered the possibility that large deletions may be underrepresented in the recovery and mutation screening assay due to the potential loss of these critical elements.

Thus, we performed long‐range PCR amplification of the mutation reporter from genomic DNA isolated from the brain and spleen tissues of 2‐ and 24‐month‐old B‐DNA and IR mice. Using the primers Far3F/Far3R, which flank the B‐DNA or IR sequence (Table S3), we expected a 1302 bp full‐length PCR product (Figures 2c, S2a). Deletions within this amplification region would result in PCR products shorter than 1302 bp, while large deletions resulting in the loss of one or both primer binding sites would prevent amplification altogether. However, only full‐length products were consistently observed in brain and spleen tissue DNA (Figure 2d,e), suggesting either the absence of deletions within the amplification range or the deletions extended beyond the detectable range due to the loss of primer binding sites.

To expand the detection range, we used primers Far3F/pBR322oriR encompassing the B‐DNA or IR sequence (Table S3), producing a 4089 bp full‐length PCR product (Figure 2f). Validation of this system using the parent p2RT reporter alone and in combination with a verified large deletion mutant confirmed the ability to detect deletions exceeding 3 kb (Figure S2b). Using this approach, we detected predominantly full‐length products and a slight age‐associated increase in the proportion of shorter products in the brain tissue of B‐DNA mice. In contrast, IR mice, particularly at 24 months, exhibited a higher frequency of truncated PCR products (< 1 kb), indicating large deletions exceeding 3 kb (Figure 2g). Analysis of spleen genomic DNA showed mostly full‐length products in both B‐DNA and IR mice, though an age‐dependent increase in shorter fragments was evident, particularly in 24‐month‐old IR mice (Figure 2h). While large deletions in the spleen were less frequent than in the brain, their presence further supports age‐associated mutagenesis driven by IR sequences. Together, the PCR results are consistent with the mutation frequency data and highlight a more pronounced effect in the aged brain tissue, likely due to its low cell turnover.

The formation of cruciform structures by short, inverted repeats, such as the 28 bp IR sequence studied here, generally requires substantial negative supercoiling density (approximately −0.05) to overcome the energetic barrier for structure formation in vivo. Negative supercoiling can arise from several cellular processes, including transcriptional activity (Achar et al. 2020; Dayn et al. 1992; G. Wang et al. 2006) replication stress (Kaushal et al. 2019), nucleosome remodeling (Miura et al. 2019), and environmental factors (Dayn et al. 1991). We have previously shown using lacZ reporter constructs in COS‐7 cells that transcription through alternate DNA structure‐forming sequences can drive mutagenesis (G. Wang et al. 2006). Consistent with these findings, the reporter used in this study is driven by a constitutive promoter, and transcription‐associated supercoiling and nucleosome exclusion represent plausible mechanisms underlying cruciform formation at the IR locus. Although replicative stress in proliferative tissues may further enhance negative supercoiling, the observation of mutations in post‐mitotic neurons where DNA replication is minimal highlights transcription as the more likely driver of local topological changes. Future studies aimed at directly characterizing transcriptional activity and chromatin topology at the reporter locus in relevant tissues will help clarify the physiological contexts that support cruciform extrusion and mutagenesis.

Several endonucleases, junction‐resolvases, transcription factors, and DNA repair proteins have been shown to interact with cruciform DNA structures or IRs, but the extent of their roles in the mutagenic processing of short IRs remains to be determined (Bowater et al. 2022; Brazda et al. 2011; Lu et al. 2015). In an in vitro pilot study we showed that short IR–induced mutagenesis proceeds independently of classical NER and MMR pathways but is significantly elevated when CtIP or MRE11 proteins are depleted, indicating that these proteins normally act to suppress IR‐associated genomic instability (Mandke and Vasquez 2024). Building on these findings, we plan to expand our in vitro screening to additional DNA repair proteins to prioritize candidate pathways most relevant to IR‐induced instability. This approach will guide in vivo studies using IR transgenic mice crossed with DNA repair–deficient strains to assess how specific repair deficiencies and aging influence IR‐induced mutagenesis.

While several plausible mechanisms may underlie the tissue‐specific differences we observed, future studies examining tissue‐ and sex‐specific differences will further clarify the molecular pathways driving IR‐induced mutagenesis. As this is a preliminary study primarily focused on characterizing age‐associated genomic instability at short, inverted repeats (IRs), we did not assess organismal aging phenotypes. Future work may also include a comprehensive evaluation of age‐associated traits, such as frailty indices, hematopoietic and immune system alterations, and metabolic markers, to validate the relevance of this model to physiological aging.

In summary, we have established and characterized a transgenic mouse model to examine the complex dynamics of age and short IR‐induced genomic instability. Our findings demonstrate that short IRs are inherently mutagenic, providing the first evidence linking age to enhanced IR‐mediated mutagenesis. Collectively, these findings advance our understanding of DNA repeat‐mediated genomic instability and aid in developing therapeutic strategies for aging‐related diseases.

## 2 Methods

Please see Supporting Information.

## Author Contributions

Conceptualization by K.M.V., G.W., P.K., and P.M.; Methodology by P.K., P.M., G.W., and K.M.V.; Investigation by P.K. and P.M.; Formal analysis by P.K. and P.M.; Writing by P.K., P.M., and K.M.V.; Editing by P.K., P.M., G.W., and K.M.V.; Supervision by G.W. and K.M.V.; Funding acquisition by K.M.V.

## Conflicts of Interest

The authors declare no conflicts of interest.

## Supporting information


**Figure S1:** Mutation reporter DNA recovery is outlined in steps 1–5. (1) The mutation reporter is separated from mouse genomic DNA through restriction digestion with SpeI (black arrows), (2) Selective recovery is then performed using magnetic beads coated with lacI‐lacZ fusion protein that bind specifically to lacI binding sites (brown circles) on the mutation reporter, (3) The mutation reporter is eluted in its linearized form using IPTG, (4) The mutation reporter is re‐circularized using T4 DNA ligase, and (5) transformed into *E. coli* DH10β cells for mutation screening. Blue circles represent wild‐type colonies, and white circles represent mutant colonies. Amp^R^, ampicillin resistance; IPTG, isopropyl ß‐D‐1‐thiogalactopyranoside; Neo^R^, neomycin resistance; Ori, origin of replication.
**Figure S2:** Validation of PCR products using Far3F/Far3R primers generating a 1302 bp full‐length PCR product. (b) FAR3F/pBR322OriR primers on the parent p2RT reporters. L, 1 kb ladder; 1, FVB mouse genomic (negative control); (2) FVB mouse genomic DNA + B‐DNA mutation reporter (mimics B‐DNA mouse genome); (3) FVB mouse genomic DNA + IR mutation reporter (mimics IR mouse genome); (4) FVB mouse genomic DNA + a known mutation reporter + the known mutation reporter with a large deletion (positive control); (5) FVB mouse genomic DNA + the known mutation reporter with a large deletion.
**Table S1:** Mutation spectra of mutation‐reporter DNA from brain tissue. Different types of point mutations (transitions, transversions, and deletions) and deletions (small and large) are presented with their respective base pair position on the mutation reporters rescued from B‐DNA mice aged to 2 months (N = 4, sequences analyzed: 28) and 24 months (N = 4, sequences analyzed: 17); IR mice aged to 2 months (N = 4, sequences analyzed: 15) and 24 months(N = 4, sequences analyzed: 7).
**Table S2:** Mutation spectra of mutation‐reporter DNA from spleen tissue. Different types of point mutations (transitions, transversion, and deletions) and deletions (small and large) are presented with their respective base pair position on the mutation reporters rescued from B‐DNA mice aged to 2 months (N = 4, sequences analyzed: 14) and 24 months (N = 4, sequences analyzed: 14); IR mice aged to 2 months (N = 4, sequences analyzed: 23) and 24 months (N = 4, sequences analyzed: 30).
**Table S3:** Primers used for PCR analysis.

## Data Availability

Data available on request from the authors.
