# Supplementary material for: Aging Increases Short, Inverted Repeat‐Mediated Genomic Instability In Vivo
Source: Aging Cell. 2025 Sep 30;24(12):e70250. doi: 10.1111/acel.70250 (PMC12686595; doi:10.1111/acel.70250)
Supplement: Supplementary file 1 — Figure S1: Mutation reporter DNA recovery is outlined in steps 1–5. (1) The mutation reporter is separated from mouse genomic DNA through restriction digestion with SpeI (black arrows), (2) Selective recovery is then performed using magnetic beads coated with lacI‐lacZ fusion protein that bind specifically to lacI binding sites (brown circles) on the mutation reporter, (3) The mutation reporter is eluted in its linearized form using IPTG, (4) The mutation reporter is re‐circularized using T4 DNA ligase, and (5) transformed into E. coli DH10β cells for mutation screening. Blue circles represent wild‐type colonies, and white circles represent mutant colonies. AmpR, ampicillin resistance; IPTG, isopropyl ß‐D‐1‐thiogalactopyranoside; NeoR, neomycin resistance; Ori, origin of replication. Figure S2: Validation of PCR products using Far3F/Far3R primers generating a 1302 bp full‐length PCR product. (b) FAR3F/pBR322OriR primers on the parent p2RT reporters. L, 1 kb ladder; 1, FVB mouse genomic (negative control); (2) FVB mouse genomic DNA + B‐DNA mutation reporter (mimics B‐DNA mouse genome); (3) FVB mouse genomic DNA + IR mutation reporter (mimics IR mouse genome); (4) FVB mouse genomic DNA + a known mutation reporter + the known mutation reporter with a large deletion (positive control); (5) FVB mouse genomic DNA + the known mutation reporter with a large deletion. Table S1: Mutation spectra of mutation‐reporter DNA from brain tissue. Different types of point mutations (transitions, transversions, and deletions) and deletions (small and large) are presented with their respective base pair position on the mutation reporters rescued from B‐DNA mice aged to 2 months (N = 4, sequences analyzed: 28) and 24 months (N = 4, sequences analyzed: 17); IR mice aged to 2 months (N = 4, sequences analyzed: 15) and 24 months(N = 4, sequences analyzed: 7). Table S2: Mutation spectra of mutation‐reporter DNA from spleen tissue. Different types of point mutations (transitions, transversion [file ACEL-24-e70250-s001.docx]

**Supplementary Figures and Tables:**


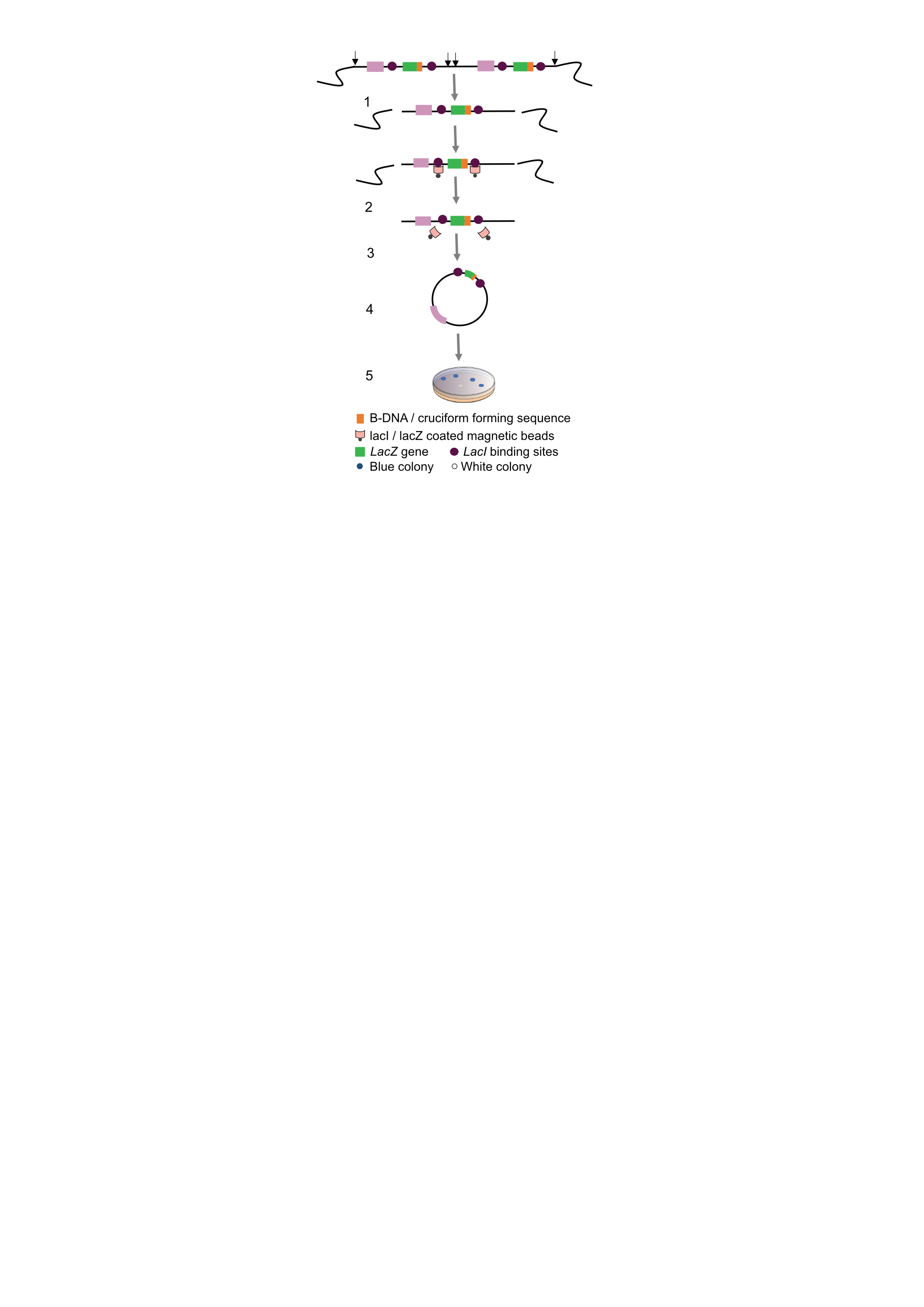


**Supplementary Figure 1.** **Mutation reporter DNA recovery** is outlined in steps 1-5. 1: The mutation reporter is separated from mouse genomic DNA through restriction digestion with SpeI (black arrows), 2: Selective recovery is then performed using magnetic beads coated with lacI-lacZ fusion protein that bind specifically to lacI binding sites (brown circles) on the mutation reporter, 3: The mutation reporter is eluted in its linearized form using IPTG, 4: The mutation reporter is re-circularized using T4 DNA ligase, and 5: transformed into E. coli DH10β cells for mutation screening. Blue circles represent wild-type colonies, and white circles represent mutant colonies. Amp^R^ Ampicillin resistance, Neo^R^ Neomycin resistance, Ori Origin of replication, IPTG Isopropyl ß-d-1-thiogalactopyranoside.

**Supplementary Figure 2.** Validation of PCR products using Far3F/Far3R primers generating a 1302 bp full-length PCR product. **b** FAR3F/pBR322OriR primers on the parent p2RT reporters. L, 1 kb ladder; 1, FVB mouse genomic (negative control); 2, FVB mouse genomic DNA + B-DNA mutation reporter (mimics B-DNA mouse genome); 3, FVB mouse genomic DNA + IR mutation reporter (mimics IR mouse genome); 4, FVB mouse genomic DNA + a known mutation reporter + the known mutation reporter with a large deletion (positive control); 5, FVB mouse genomic DNA + the known mutation reporter with a large deletion.


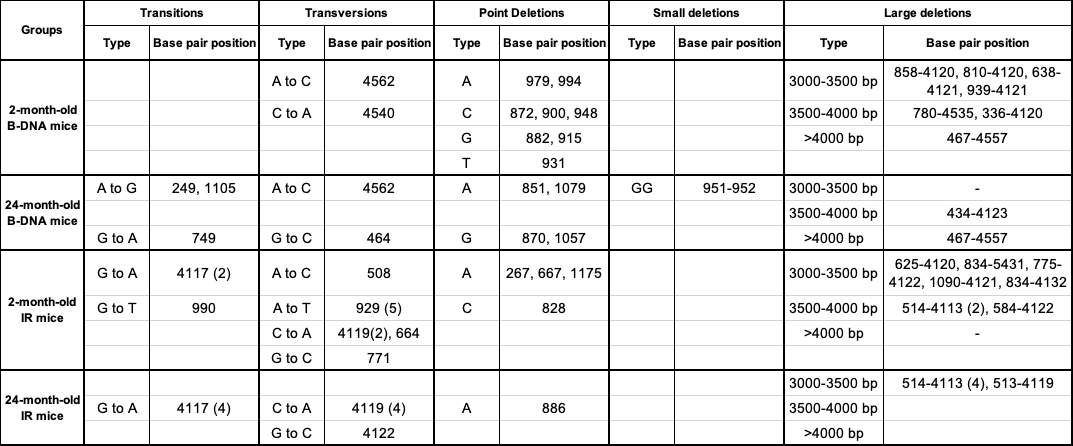


**Supplementary Table 1: Mutation spectra of mutation-reporter DNA from brain tissue.** Different types of point mutations (transitions, transversions, and deletions) and deletions (small and large) are presented with their respective base pair position on the mutation reporters rescued from B-DNA mice aged to 2 months (N=4, sequences analyzed: 28) and 24 months(N=4, sequences analyzed: 17); IR mice aged to 2 months (N=4, sequences analyzed: 15) and 24 months(N=4, sequences analyzed: 7).


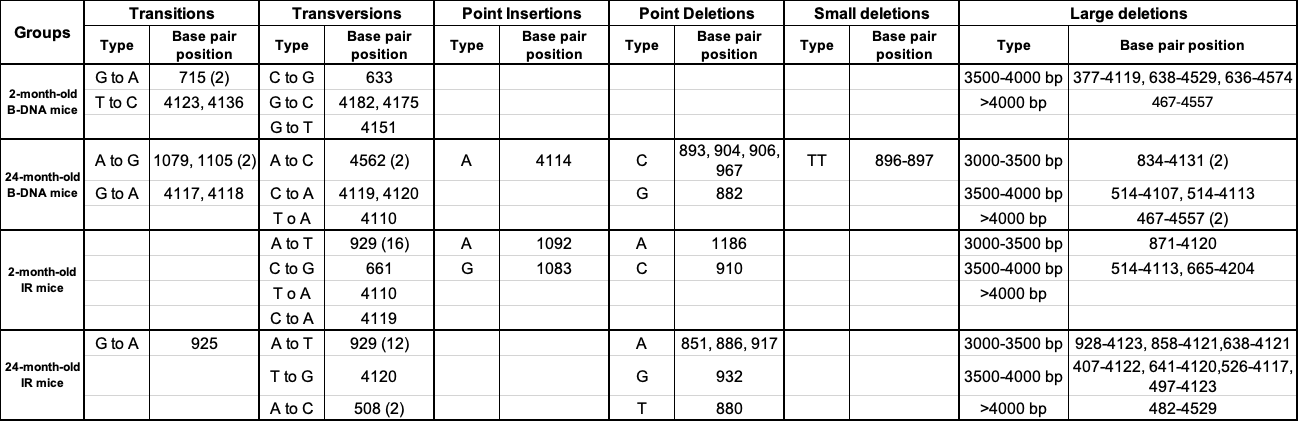


**Supplementary Table 2: Mutation spectra of mutation-reporter DNA from spleen tissue**. Different types of point mutations (transitions, transversion, and deletions) and deletions (small and large) are presented with their respective base pair position on the mutation reporters rescued from B-DNA mice aged to 2 months (N=4, sequences analyzed: 14) and 24 months(N=4, sequences analyzed: 14); IR mice aged to 2 months (N=4, sequences analyzed: 23) and 24 months(N=4, sequences analyzed: 30).

| **Primer name** | **Sequence (5’🡪3’)** |
| --- | --- |
| Far3F | GGTGATGACGGTGAAAACCT |
| Far3R | CCATAAAACCGCCCAGTCTA |
| pBR322oriR | GGGAAACGCCTGGTATCTTT |

**Supplementary Table 3: Primers used for PCR analysis.**

**Methods**

**Transgenic mouse model**

A transgenic mouse model with a chromosomally integrated recoverable mutation reporter was developed, following a similar to the one described by Wang G. et al. (2008) ([Wang et al., 2008](#_ENREF_1)). Briefly, a 29-bp GC-rich inverted repeat (IR) sequence capable of adopting a cruciform structure was cloned into the *LacZ* gene of the mutation reporter, p2RT. In addition, a B-DNA-forming sequence was cloned into the *LacZ* gene to serve as a control. In addition to elements for replication and antibiotic resistance, the p2RT reporter contains two lacI binding sites and specific restriction digestion enzyme sites for its recovery from the mouse chromosomes([Wang et al., 2009](#_ENREF_2)).

Linearized B-DNA and short IR-containing reporter constructs were microinjected into fertilized FVB/N mouse oocytes and implanted into pseudo-pregnant mice, generating B-DNA and IR transgenic mice, respectively. The presence of the cruciform-forming or B-DNA control sequences in the genomes of the mice was confirmed by genotyping genomic DNA from mouse tail snips. B-DNA and IR founder mice were subsequently crossed with wild-type FVB/N mice to obtain the F1 generation. Another round of genotyping and mutation reporter copy number estimation using real-time qPCR was performed on F1 pups. The mice with the highest integrated reporter copy numbers were selected for this study.

**Aging study**

Groups of male and female B-DNA and IR mice (n=4) were aged for 2 and 24 months. Mice were regularly monitored for signs of pain and distress, external physical appearance, and behavioral changes. After the study, the mice were euthanized by CO_2_ asphyxiation, and brain and spleen tissues were collected and flash-frozen in liquid nitrogen.

The mice were housed in polycarbonate cages (5 mice/cage) on autoclaved bedding and provided with sterile water and chow diet ad libitum. The housing environment was maintained at 20°C to 22°C, with a relative humidity of 60% to 70%, and a 12/12-hour light/dark cycle. All procedures were conducted in compliance with an approved Institutional Animal Care and Use Committee protocol (AUP-2016-00286).

**Genomic DNA extraction**

Brain and spleen tissue samples (25-30 mg) were crushed (while on dry ice) using a clean razor blade and incubated overnight (O/N) at 50°C in 0.5 mL of lysis buffer (5 mM EDTA, 20 mM Tris-HCl pH 8.0, 400 mM NaCl, and 1% SDS) supplemented with 100 μL of 20 mg/mL proteinase K and 20 μL of 20 mg/mL RNAse A. The following day, lysed samples were subjected to two sequential extractions with one volume of phenol/chloroform and precipitated O/N at -20°C with 10% 3 M sodium acetate and 2.5 volumes of ethanol. The precipitated DNA was collected by centrifugation, air-dried, and resuspended in 200 μL of 10 mM Tris-HCl, pH 8.0. DNA concentration was measured using a NanoDrop™ 2000/200c spectrophotometer (Thermo Fisher Scientific, Waltham, MA) and stored at 4°C until use.

**Mutation reporter recovery from mouse genomic DNA**

The linearized mutation reporter cassettes were recovered from mouse genomic DNA as previously described ([Wang et al., 2008](#_ENREF_1); [Wang et al., 2009](#_ENREF_2)) with a few modifications **(Figure 1c)**. Briefly, 70-80 μg of the brain or spleen genomic DNA was digested twice in a 400 μL reaction containing SpeI-HF restriction enzyme (1U/μg) and 1X cut smart buffer (NEB, Ipswich, MA) at 37°C for 4 hours, followed by phenol/chloroform extraction and ethanol precipitation. The digested DNA was dissolved in 100 μL of 1X binding buffer (10 mM Tris-HCl, pH 6.8, 1 mM EDTA, 10 mM MgCl_2_, 5% glycerol) and incubated with 80 μL of lacI-lacZ coated sheep anti-mouse IgG Dynabeads (Thermo Fisher Scientific, Baltics UAB) at 37°C for 1 hour with rotation. The beads were pelleted using a magnetic particle concentrator and washed thrice with 250 μL 1X binding buffer to remove mouse genomic DNA. The mutation reporter DNA was eluted by resuspending the beads in 100 μL of water, 75 μL of IPTG elution buffer (10 mM Tris-HCl, pH 7.5, 1 mM EDTA, 125 mM NaCl), 20 μL of NEB buffer 2 (NEB, Ipswich, MA), and 5 μL of 25 mg/mL IPTG, followed by incubation at 37°C for 1 hour with rotation. The supernatant containing the reporter DNA was collected, and ethanol precipitated with 20 µg of glycogen overnight at -20°C.

The recovered reporter DNA was circularized by incubating with blunt TA ligase (NEB, Ipswich, MA) at 25°C for 45 minutes, followed by phenol/chloroform extraction and ethanol precipitation with 20 µg of glycogen overnight at -20°C. The following day, the ligated mutation reporter plasmid was pelleted by centrifugation, air-dried, resuspended in 8 μL of 10 mM Tris-HCl (pH 8.0), and stored at 4°C until use.

**Mutation screening by blue-white mutagenesis assay**

For this assay, 2 μL of the recovered reporter DNA was mixed with 25 μL of cytosine methylation resistant deficient DH10β *Escherichia coli* *(E. coli)* electrocompetent cells (Thermo Fisher Scientific, Waltham, MA) and transformed using BioRad GenePulser® II (1.7 kV, 200 Ω, and 25 μF). Following recovery according to the manufacturer's instructions, the cells were plated on LB agar plates containing 100 μg/mL carbenicillin, 200 μg/mL X-Gal, and 400 μg/mL IPTG (XIC) and incubated at 37°C O/N. Bacterial colonies with mutations in the *LacZ* gene appeared white, while those without mutations appeared blue on X-gal plates. Mutation frequency was calculated as a ratio of white colonies to the total number of colonies (blue + white). To ensure accuracy, more than 20,000 colonies were counted per mouse tissue, with mutations standardized to 10,000 colonies for uniform reporting.

**Mutant characterization**

Approximately 30-40 mutant colonies per age group for the brain and spleen tissues of the B-DNA and IR mice were randomly selected and streaked on LB agar X-gal plates and incubated at 37°C O/N. The following day, single white colonies were inoculated into 5 mL of LB broth containing 100 μg/mL carbenicillin and incubated at 37°C O/N with rotation. A single blue colony per group was used as a wild-type (non-mutant) reference control for comparison. Reporter DNA was isolated from overnight cultures using the QIAprep spin miniprep kit (Qiagen, Germantown, MD) and analyzed via Sanger sequencing.

**Mutation spectra analysis**

Mutant sequences were compared with the respective B-DNA or IR reporter sequences using nucleotide BLAST. The mutants within the *LacZ* reporter gene were classified into two categories: point mutations and large deletions. Point mutations included transitions, transversions and, single base pair insertions, and deletions. Deletions <30 bp were categorized as small deletions, and those >30 bp were included as large deletions. The contribution of each mutation type to the overall mutation frequency was determined by counting mutants with one dominant mutation per mouse sample, prioritizing large deletions over point mutations. The proportion of each dominant mutation was calculated as a percentage of the total mutants and multiplied by its corresponding mutation frequency to represent the contribution of each mutation type to the overall mutation frequency.

**Mutation screening by PCR**

Mutants were also subjected to PCR analysis to identify events potentially in the blue-white screen due to the deletion of *lacI/lacZ* binding sites. For this, 100- 200 ng of purified genomic DNA containing the linearized reporter was amplified with 0.25 µM primers **(Supplementary Table 3)** and 1X GoTaq master mix (Promega Corporation, Madison, WI) in a 20 µL reaction. Positive controls included linearized parent p2RT B-DNA, IR, or a known mutation reporter plasmid combined with FVB mouse genomic DNA matching the reporter copy number observed in the transgenic mouse model. For large deletion mutants, a positive control was prepared by combining FVB mouse DNA with a known mutation reporter with a large deletion and the parent reporter plasmid at a ratio reflecting the in vivo propensity. DNA from FVB mice without the reporter DNA served as a negative control. A standard PCR amplification program was used, with modifications to the exception of the extension step: 72°C 60 seconds for 25 cycles for the primer pair Far3F/Far3R and 72°C 4 minutes 30 seconds for 29 cycles for the primer pair Far3F/ pBR322oriR. The PCR products were resolved on 1% agarose gels in TBE buffer at 80 V, stained with SYBR Gold, and visualized using the ChemiDoc imaging system (Bio-Rad Laboratories, Hercules, CA).

**Statistical analysis**

Statistical analyses of mutation frequencies and spectra were performed using Graphpad Prism v9. Data normality was assessed using the Shapiro-Wilk test, and outliers were identified and removed using the ROUT method. Results were presented as mean ± SEM for mutation frequency and mutation spectrum (dependent variables). Two-way ANOVA was performed with age (2-months *vs.* 24-months) and sequence type (B-DNA *vs.* IR) as independent variables. Interaction effects and the main effects of age and sequence type were reported as F-test values and corresponding p-values. Post hoc analysis was conducted using the Šidák multiple comparisons test, with statistical significance set p<0.05. Significance levels were denoted as *p<0.05, **p<0.01, ***p<0.001, and ****p<0.0001.

**Supplementary References**

Wang, G., Carbajal, S., Vijg, J., DiGiovanni, J., & Vasquez, K. M. (2008). DNA structure-induced genomic instability in vivo. *J Natl Cancer Inst*, *100*(24), 1815-1817. <https://doi.org/10.1093/jnci/djn385>

Wang, G., Zhao, J., & Vasquez, K. M. (2009). Methods to determine DNA structural alterations and genetic instability. *Methods*, *48*(1), 54-62. <https://doi.org/10.1016/j.ymeth.2009.02.012>
